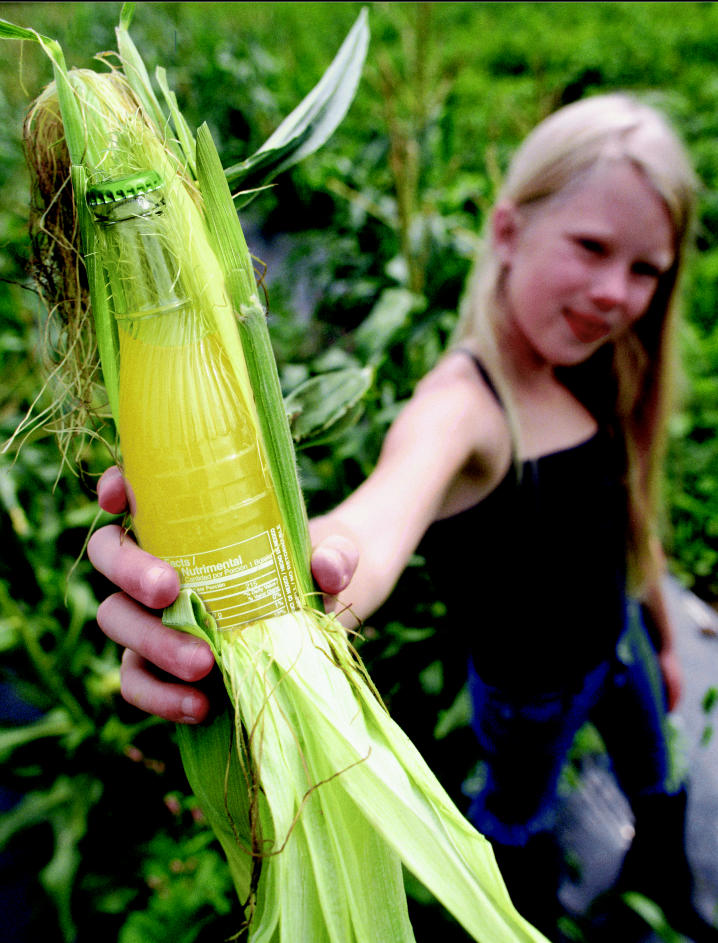# The Fat of the Land: Do Agricultural Subsidies Foster Poor Health?

**DOI:** 10.1289/ehp.112-a820

**Published:** 2004-10

**Authors:** Scott Fields

## Abstract

Ever since the Great Depression, American farmers have been the beneficiaries of a medley of subsidies and support programs meant to stabilize crop prices, keep farmers farming, and provide U.S. families with an affordable, reliable supply of food. But these programs may have had an unintended side effect. Rather than keep Americans healthy, critics say, these policies have contributed to today’s obesity pandemic and other nutrition problems as well.

Writing in the 2004 *Annual Review of Nutrition*, James Tillotson, a professor of food policy and international business at Tufts University, argues that U.S public policy encourages obesity at the expense of sound nutritional practices. “You have a whole régime here that’s worked to increase agricultural efficiency,” Tillotson says. And what U.S. farmers are most efficient at producing, he says, are just a few highly subsidized crops—wheat, soybeans, and especially corn.

Support for these few crops, critics say, has compelled farmers to ignore other crops such as fruits, vegetables, and other grains. The market is flooded with products made from the highly subsidized crops, including sweeteners in the form of high-fructose corn syrup (HFCS), fats in the form of hydrogenated fats made from soybeans, and feed for cattle and pigs. This flood, in turn, drives down the prices of fattening fare such as prepackaged snacks, ready-to-eat meals, fast food, corn-fed beef and pork, and soft drinks. Worse yet, some scientists say, paltry support for foods other than these staples increases the contrast between prices of fat-laden, oversweetened foods and those of healthier alternatives, offering poor folks little choice but to stock their pantries with less nutritious foods.

Hogwash, say other researchers and agricultural industry professionals, who cite a number of other changes that are making Americans fat. Less physical activity is one major lifestyle change that has led to more obesity. Longer work weeks and more two-worker households both mean less time for nutritious home-cooked meals. They also mean more “latchkey children” left home alone from the time they leave school until their parents get home from work—children who tend to be less active and eat more fattening snacks. Technological innovations have contributed as well—for example, advances in cutting and peeling technology, freezing technology, coating technology (McDonald’s fries have a coating of sugar and beef flavoring), transportation technology, and cooking technology have put fattening french fries on nearly every restaurant menu in America. Persuasive television commercials and just plain personal taste are also making Americans fat, they contend.

Even if price supports were eliminated entirely, says Larry Mitchell, CEO of the American Corn Growers Association in Washington, D.C., prices for subsidized commodities wouldn’t increase significantly, and they might even drop. Furthermore, says Sam Willett, senior director of public policy for the National Corn Growers Association, also in Washington, demand for products, not agricultural subsidies, determines what farmers choose to grow. “Connecting farm programs to obesity is quite a leap,” he says. “When you examine the data, it doesn’t support the theory. The fact is, farmers are capturing less and less of the total food dollar.”

Any way you look at it, the number of factors involved makes it hard to encapsulate the relationship between farm support and obesity in a neat cause-and-effect equation. Even the U.S. Department of Agriculture (USDA)—which administers both the agricultural subsidy programs and a host of nutrition research and education programs—has put scant, if any, thought into a possible relationship between the two, says Elizabeth Frazao, a nutrition scientist for the USDA Economic Research Service. Department spokeswoman Jean Daniel says USDA research indicates simply that overweight children and adults are eating too much and not getting enough physical activity.

## Support for Farmers

American farm subsidies have evolved over the last 80-plus years from an emergency stopgap into an apparently inviolable institution that, despite efforts to scale back, is bigger than ever. The first American agricultural assistance programs started in the 1920s to address ramped-up growing patterns that farmers had developed in support of the World War I effort. When the war ended, farmers continued to grow crops at a record pace. The result was a glut of produce followed soon by plummeting prices, which the Agricultural Credits Act of 1923 was unable to stop.

Since then, the U.S. government has employed a chain of programs that at times have attempted to manage what and how much American farmers produce. As early as 1929 the government bought cotton and grains on the open market when production outstripped demand in an attempt to stabilize prices. That just encouraged farmers to grow more. Later techniques included fixing quotas for certain farm products, removing surplus products from the marketplace, and paying farmers not to plant crops that were flooding the market.

According to Richard Wiles, a senior vice president for the nonprofit Environmental Working Group, these programs have become entrenched in America’s heartland. Although farm subsidies began to taper off in the early 1960s, during the first term of the Nixon administration an unfortunate convergence of a poor growing year and an agreement to sell millions of bushels of grain to the Soviet Union caused shortages and a spike in prices. In response, the government developed a suite of programs meant to increase production. The result, Wiles says, was a surplus of basic commodities—primarily wheat, corn, soybeans, and cotton—and falling prices for these products on the open market. In 1996 an attempt was made to eliminate subsidies altogether. This so-called Freedom to Farm Act eliminated crop subsidies, but instead gave farmers fixed amounts of money based on what they had grown in earlier years.

According to Wiles, however, the act was fatally flawed. “It grandfathered everybody who received subsidies at that time so that they could get subsidies forever, whether or not they grow anything. It turned the commodity payments into commodities themselves that could be passed around, sold, and traded.”

By 2000 these fixed payments had reached $22 billion, about three times the pre-reform level of 1996, according to the 2002 report *Landowners’ Riches: The Distribution of Agricultural Subsidies* by Ohio State University agricultural economist Barry K. Goodwin and colleagues. The 2002 Farm Bill abandoned this attempt to eliminate subsidies and reduce farm payments. Instead, says *Landowners’ Riches*, it is scheduled to distribute about $190 billion by 2012, an increase of about $72 billion when compared to the programs it replaced. Supporters call this provision a vital safety net for America’s most vulnerable workers—small family farmers with few resources. Some critics, on the other hand, call it welfare that benefits huge agricultural corporations—giant farms, grain brokers, food processors, fast-food chains, and prepackaged food companies—more than family farms.

## From Farm Fields to Grocery Bills

This support may indeed drive down the price of commodities such as corn, wheat, and soybeans. To Marion Nestle, a professor of nutrition, food studies, and public health at New York University, that’s one of the reasons the relationship between agricultural subsidies and obesity is clear. Because prices of these staples are low, so are those of HFCS, hydrogenated fats, and corn-fed meats. And the cheapest way to make foods taste good, she says, is to add sugars and fat.

Compounding the problem, says Barry Popkin, a professor of nutrition at the Carolina Population Center of the University of North Carolina at Chapel Hill, is that fattening foods are supported whereas healthy fare isn’t. “We put maybe one-tenth of one percent of our dollar that we put into subsidizing and promoting foods through the Department of Agriculture into fruits and vegetables,” he says. As a result, the price gap between high-sugar, high-fat foods and more nutritionally valuable fruits and vegetables is artificially large. That means in supermarkets and restaurants, red meats, sugar-and fat-loaded products, and fast foods not only appear to be the best buys but in proportion to even moderate salaries are downright cheap. The proportion of income required to buy food in the United States is among the lowest in the world and has declined steadily since the 1950s, according to the USDA. If anything, the 2002 Farm Bill will result in record crops planted on even fewer acres than under previous support programs, Willett says.

A shopping cart filled with inexpensive food rolls right to an overweight population, says Darius Lakdawalla, an economist at the RAND Corporation and the National Bureau of Economic Research who investigates trends in U.S. obesity. “One of the things we’ve looked at was simply the falling price of food. The price of food has fallen a lot over the past couple of decades. According to our estimates, declining food prices can account for as much as half of the increase in obesity that we’ve seen,” he explains. “In a sense it’s a very simple explanation. People face cheaper food. They eat more. And they weigh more.”

The very poorest American people, says Lakdawalla, are undernourished and thinner than the general population. But if you exclude the poorest of the poor, obesity is associated with poverty. One reason is that the fattening foods found at convenience stores and fast-food restaurants are the cheapest and sometimes the only available foods in poor neighborhoods, according to Thomas Robinson, an associate professor of pediatrics and medicine at the Stanford School of Medicine Prevention Research Center. A poor, overweight person therefore isn’t necessarily a completely nourished person, says Lakdawalla. Furthermore, poorer people can’t afford health clubs and may live in neighborhoods in which it is too dangerous to exercise outside. And because poverty is inversely related to education, poor people may be unaware of sound nutritional practices.

## The Effect on Children

As the American obesity pandemic has gathered momentum, the hazards of obesity—heart problems, diabetes mellitus, some cancers, skeletal and musculature stress, shortened life expectancy—have been much discussed in the popular press. Less well understood is the relationship between consuming too many calories and an absence of some essential minerals and vitamins, especially in children.

“Children who are obese or overweight are actually also often lacking the appropriate nutrients,” Lakdawalla says. “It’s called ‘mis-nourishment’ rather than ‘malnourishment.’” These improperly nourished children—who as of 1992 numbered about 12 million in the United States alone, according to a February 1996 *Scientific American* article—can encounter serious physical and mental development problems, such as stunted growth and cognitive impairment, says J. Larry Brown, executive director of the Brandeis University Center on Hunger and Poverty and coauthor of the *Scientific American* article.

We put maybe one-tenth of one percent of our dollar that we put into subsidizing and promoting foods through the Department of Agriculture into fruits and vegetables.

Barry Popkin, Carolina Population Center, UNC-CH

Shanthy Bowman, a nutrition scientist for the USDA Agricultural Research Service, says department research shows that when children eat foods that contribute to obesity, they miss out on the nutrients found in healthier foods. Bowman and colleagues at Harvard University reported n the January 2004 issue of *Pediatrics* how eating fast food affects the quality of children’s diets. On any given day, about 30% of the study’s 6,200 children aged 2–19 consumed some fast food. On those days they took in about 187 extra calories, more energy per gram of food (which generally translates to less dietary fiber), more fat, more carbohydrates, more added sugars, less milk, and fewer fruits and vegetables. Fast foods give children practically nothing in the way of fruits, vegetables (not counting potatoes), or milk, Bowman says.

Many of the empty calories children are taking in come from sweetened beverages, largely soft drinks, which in American homes are increasingly displacing milk and contributing to calcium deficiencies, Bowman says. Between 1965 and 1996, adolescents’ milk consumption decreased by 36% as soft drink consumption increased by 287% in boys and 224% in girls, according to research by Popkin and colleagues published in the July 2000 issue of *Archives of Disease in Childhood*. People who consume more than 18% of their calories in added sugars (and U.S. consumption of added sugars increased 28% between 1982 and 1997) have lower-than-normal levels of essential micronutrients, especially vitamin A, vitamin B_12_, folate, magnesium, and iron, Bowman says.

## HFCS: A Double-Edged Sword

In America, soft drinks are sweetened with HFCS. (In Europe beet sugar is used in soft drinks; HFCS is not allowed in order to protect European beet farmers.) Until a few decades ago, most American foods were sweetened with cane sugar from warm climates or, less often, beet sugar grown domestically. In the late 1960s, however, Japanese scientists developed a way to use enzymes to convert cornstarch into HFCS, which is sweet enough to replace other types of sucrose-based sugars. Since then, HFCS has been a success story for corn growers, but—says George Bray, a professor of nutrition at Louisiana State University—a tragedy for American health.

Between 1970—just after HFCS was developed—and 1990, consumption of HFCS in the United States increased 1,000%, according to a commentary published in the April 2004 issue of the *American Journal of Clinical Nutrition* by Bray, Popkin, and colleague Samara Joy Nielsen. It now represents 40% of the non-calorie-free sweeteners added to U.S. foods and is virtually the only source of sweeteners for soft drinks. It has also worked its way into baby food, fruit drinks, ketchup, yogurt, candies, cakes, muffins, and too many other products to count. On average, Bray says, Americans over age 2 consume at least 132 calories of HFCS per day—and that’s a conservative estimate. Americans who are in the top 20% of sweetened product consumers take in about 216 calories a day from HFCS.

It is no coincidence, Bray says, that as HFCS’s sales figures have increased, American waistlines have kept pace. HFCS is cheap, which has allowed for 25¢ snack cakes, 60¢ candy bars, and—especially—bargain-priced, giant-sized soft drinks in convenience stores, at restaurants, and on grocery store shelves.

Bray says the human body processes fructose differently than it does glucose. Glucose triggers the pancreas to release insulin, suppressing appetite. Fructose, however, is processed only in the liver, so no insulin is released. As a result, he says, people are more likely to habitually overindulge in HFCS-sweetened products. (Nestle says, however, that the percentage of fructose is the same in HCFS and cane or beet sugar—about 50%. Although there are small variations in the fructose content between the types of sugar, she says, they are not enough to affect how the body reacts to them.)

Most significantly, according to Bray, HFCS products just taste sweeter than foods made with cane or beet sugar. That trains people to expect ever-increasing levels of sweetness. Children, especially, learn quickly to crave HFCS. “We may be damaging the neuronal circuitry in the brain during this highly plastic period of development,” he says. He adds that soft drinks are especially troublesome because experimental research in the June 2000 issue of the *International Journal of Obesity and Related Metabolic Disorders* demonstrated that people will consume more calories when sweetened products are offered as liquids than when offered as solids. About two-thirds of the HFCS consumed in the United States is in beverages.

Agricultural interests stand by their product, however. The nonprofit Corn Refiners Association issued a 25 March 2004 press release in response to Bray and Popkin’s 2004 commentary, which was titled “HFCS Is *Not* a Unique Contributor to Obesity.” The release stated, “The facts are simple . . . HFCS and table sugar are indistinguishable to the human body; . . . HFCS is safe to consume and can be part of a healthy, balanced diet.” The association declined to comment further for this article.

## The Bottom Line

HFCS’s market success may be at least partly a result of two complementary government policies. Farm subsidies may reduce its cost, and tariffs plus quota restrictions on imports of foreign sugar make it a better buy than alternatives. But even eliminating farm subsidies entirely wouldn’t affect how much soda pop people drink, how many cupcakes they snack on, or even how much meat they eat, says Bruce Babcock, an economics professor at Iowa State University.

“We did an analysis that showed that if corn and soybeans were not subsidized, the price would rise at most by between five and seven percent,” Babcock explains. According to the unpublished analysis, which Babcock performed in June 2004 for the National Corn Growers Association, that much of an increase in the price of corn wouldn’t affect the price of HFCS because most of its cost is in manufacturing rather than raw materials, he says; it would affect other products, although again not by much. “A five- to seven-percent increase in the price of corn would lead to, at most, a one-percent increase in the price of meat,” says Babcock. “But meat consumption doesn’t respond dramatically to price. So what that would do is reduce consumption by point-three percent.”

If you raise the price of those inputs like corn and soybean oil, you have a very, very small impact on the prices consumers see when they make their food choices.

Bruce Babcock, Iowa State University

The problem with linking farm subsidies to the cost of fattening foods, Babcock says, is that farmers just don’t see much of the consumer’s food dollar. “The final prices of products—meat, bread, milk—don’t have a whole lot to do with the price of farm products,” he says. “So if you raise the price of those inputs like corn and soybean oil, you have a very, very small impact on the prices consumers see when they make their food choices.”

But if America is going to subsidize agriculture, the least it could do is subsidize healthy foods, says Richard Atkinson, a professor of medicine and nutritional sciences at the University of Wisconsin–Madison and president of the nonprofit American Obesity Association. “There are a lot of subsidies for the two things we should be limiting in our diet, which are sugar and fat, and there are not a lot of subsidies for broccoli and Brussels sprouts,” he says. “What would happen if we took away the subsidies on the sugar and fat? Probably not much. They might go up a little bit, but the cost of the food is not the actual cost of the final products. But if we’re trying to look for something political that might make a difference, try subsidizing fruit and vegetable growers so the cost is comparatively lower for better foods.”

## Figures and Tables

**Figure:**